# Coenzyme Q10 and Fish Oil Supplementation for Reducing Retinal Oxidative Stress in a Rat Model

**DOI:** 10.3390/vision7010020

**Published:** 2023-03-11

**Authors:** Faisal Siddiqui, Charles Cai, Jacob V. Aranda, Kay D. Beharry

**Affiliations:** 1 Department of Pediatrics, Division of Neonatal-Perinatal Medicine, State University of New York, Downstate Health Sciences University, Brooklyn, NY 11203, USA; 2 Department of Ophthalmology, State University of New York, Downstate Health Sciences University, Brooklyn, NY 11203, USA; 3 State University of New York Eye Institute, Brooklyn, NY 11203, USA

**Keywords:** antioxidants, coenzyme Q10, fish oil, intermittent hypoxia, oxidative stress, oxygen-induced retinopathy

## Abstract

Extremely low gestational-age neonates requiring supplemental oxygen experience intermittent hypoxia (IH) episodes, which predispose them to oxidative stress and retinopathy of prematurity. We tested the hypothesis that early supplementation with fish oil or CoQ10 confers benefits reducing the severity of IH-induced retinopathy. At birth, rat pups were exposed to two clinically relevant neonatal IH paradigms with recovery in either hyperoxia (50% O_2_) or room air (RA) between episodes for 14 days, during which they received daily oral fish oil, coenzyme Q10 (CoQ10) in olive oil (OO), or OO only (vehicle). At postnatal day 14 (*P*14), pups were allowed to recover in RA with no further treatment until *P*21. Retinas were examined at *P*14 and at *P*21. Both IH paradigms resulted in severe ocular oxidative stress and retinopathy regardless of recovery in hyperoxia or RA in the vehicle groups. Although early supplementation with fish oil was beneficial, CoQ10 provided superior benefits for reducing IH-induced oxidative stress and retinopathy. These effects were associated with lower retinal antioxidants and biomarkers of angiogenesis. The therapeutic benefits of CoQ10 suggest a potential treatment for IH-induced retinopathies. Further studies are needed to establish appropriate, safe, and effective doses for use in preterm infants.

## 1. Introduction

Retinopathy of prematurity (ROP) is a developmental vascular disorder that afflicts extremely low gestational age (<28 weeks gestation; <1250 g birth weight) neonates (ELGANs) because of incomplete retinal vascularization. ROP remains one of the major causes of childhood blindness that is potentially avoidable [1,2]. The prevalence of blindness due to ROP is about 50,000 per million births worldwide [3]. The incidence of ROP of any stage in the United States of America among ELGANs is 68% [4]. An increase in birth weight and gestational age leads to a decrease in the threshold of ROP [5]. Multiple factors play a role in ROP development, but prematurity, oxygen toxicity, intermittent hypoxia (IH), and poor postnatal growth are a few important factors [6]. Exposure to hyperoxia increases the incidence of ROP in ELGANs [7]. This is likely due to immature antioxidant systems and frequent arterial oxygen desaturations, which may resolve in hyperoxia or room air between episodes. Neonatal IH is defined as brief repetitive cycles of hypoxia lasting less than 3 min [8,9]. The combination of hyperoxia, IH, and immature antioxidant systems predispose ELGANs to the accumulation of reactive oxygen species (ROS) and oxidative damage [10].

Oxidative stress alters intracellular redox homeostasis. In the retina, altered redox status combined with suppression of vascular growth factors contributes to the severity of ROP. One of the key mechanisms of oxidative damage involves the interaction between hydrogen peroxide (H_2_O_2_) and iron. In oxidative stress, superoxide anions are the major ROS produced, which, when encountering superoxide dismutase (SOD, the first line of defense), are converted to H_2_O_2_ and oxygen. Catalase and glutathione (GSH) neutralize and detoxify H_2_O_2_. However, if allowed to accumulate due to inefficient scavenging, H_2_O_2_ can react with free iron to form the highly reactive hydroxyl radical via the Fenton–Haber–Weiss reaction [11]. Hydroxyl radicals rapidly target and oxidize lipids, proteins, and nucleic acids, causing oxidative damage via self-propagating lipid peroxidation [12]. Lipid peroxidation decreases GSH and leads to DNA impairment and membrane permeability [13]. In general, the retina is susceptible to lipid peroxidation due to its abundance of polyunsaturated fatty acids (PUFAs) in the photoreceptors, but the immature retinas of ELGANs are particularly vulnerable. In addition to immature antioxidant systems, ELGANs are deficient in omega-3 PUFAs, which make up most of the lipid structure of the retina [14]. PUFAs are anti-angiogenic and neuroprotective, and they have antioxidant properties. Their administration leads to improved growth and reductions in the severity of oxygen radical diseases in neonates, which includes ROP [15,16]. CoQ10 plays an important role as a cofactor in the electron transport chain in the mitochondria, which leads to energy production. It also has antioxidant properties and can directly scavenge free radicals and prevent membrane phospholipid peroxidation, eventually decreasing the damage from ROS [17,18]. Previous work examining the benefits of CoQ10 or fish oil for growth and retinal angiogenesis in neonatal rats exposed to a single IH paradigm showed that fish oil was more beneficial for growth [18], and both CoQ10 and fish oil did not completely prevent severe OIR [19]. Because both CoQ10 and fish oil have antioxidant properties, we asked whether the results of our findings were due to recovery in hyperoxia between episodes. In the current study, we addressed this by adding a different IH paradigm with recovery in normoxia (21% O_2_) between each IH event, and we focused mainly on antioxidants.

Based on the proven benefits of fish oil and CoQ10, we hypothesized that early supplementation with fish oil or CoQ10 during IH would confer beneficial effects on the retina, thereby leading to a reduction in the severity of IH-induced retinopathy. Our hypothesis was tested with the following objectives: (1) to demonstrate, in principle, that exposure to IH causes severe retinopathy regardless of recovery/reoxygenation in hyperoxia or room air; (2) to establish that IH-induced retinopathy is associated with oxidative stress; (3) to compare the efficacy of early supplementation with fish oil or CoQ10 during IH for reducing ocular oxidants, improving antioxidant status, and preserving retinal integrity. The primary outcome was retinal damage, and the secondary outcomes were the systemic and ocular biomarkers of oxidative stress and antioxidants.

## 2. Materials and Methods

### 2.1. Animals

All experiments were approved by the State University of New York, Downstate Medical Center Institutional Animal Care and Use Committee, Brooklyn, NY (protocol #19-10559). All animals were cared for according to the National Institutes of Health Guide for the Care and Use of Laboratory Animals. Certified infection-free, timed-pregnant Sprague Dawley rats were purchased from Charles River Laboratories (Wilmington, MA, USA) at 18 days of gestation. The animals were housed in an animal facility with a 12-h day/12-h night cycle and allowed free access to a standard laboratory diet and water.

### 2.2. Experimental Design

At birth (postnatal day 0, *P*0), newborn rats were pooled from three litters to eliminate litter differences. From this pool, nine male rats and nine female rats were chosen for each group. Pups that were >10% or <10% of the average weight were not used. Gender identification and expanded litter sizes were previously described [18,19,20]. The neonatal IH paradigms and treatment protocols were conducted as previously reported [19,20]. Supplementation occurred only from *P*0 to *P*14 via orogastric feeding needles. Age-matched controls were raised in RA from *P*0 to *P*21 and were similarly supplemented.

### 2.3. Neonatal Intermittent Hypoxia (IH) Profiles

Episodes of reoxygenation following an IH event may occur in normoxia or hyperoxia. Animals randomized to both IH paradigms were placed, with the dams, in specialized oxygen chambers attached to an oxycycler (BioSpherix, Parish, NY, USA). The details of the IH profiles were previously described [19,20]. Like preterm infants, the eyes of newborn rats are immature. Exposure to neonatal IH results in many characteristics consistent with ROP. Our model was validated and proven to reliably result in severe oxygen-induced retinopathy (OIR).

### 2.4. Supplementation

CoQ10, fish oil, or olive oil supplementation occurred only from *P*0 to *P*14 [18,20]. Pups were either studied at *P*14 to determine immediate effects or allowed to recover in RA until *P*21. Oxygen saturation before and during neonatal IH was determined as previously described [19,20].

### 2.5. Sample Collection and Processing

At euthanasia, whole eyes were collected to assess retinal damage (primary outcome). Blood, retina, choroid-retinal pigmented epithelium (RPE), and vitreous fluid were collected to assess biomarkers of oxidative stress and antioxidants (secondary outcomes). At *P*14 and *P*21, following decapitation, mixed arterial and venous blood samples were collected into sterile Eppendorf tubes and placed on ice for 30 min. Samples were centrifuged at 3500 g for 30 min at 4 °C. The resulting serum was transferred to a clean sterile Eppendorf tube and frozen at −20 °C until analysis. All samples were analyzed on the same day. For ocular samples, eyes were enucleated and rinsed in ice-cold, phosphate-buffered saline (PBS, pH 7.4) on ice. The isolation, collection, and processing of retinal and choroid-RPE samples were performed as previously described [18,19,20,21,22]. Retinas were separated from the underlying choroid-RPE, placed in sterile Lysing Matrix D 2.0 mL tubes containing 1.4 mm ceramic spheres (MP Biomedicals, Santa Ana, CA, USA), and snap-frozen in liquid nitrogen prior to freezing at −80 °C until analysis. Choroid-RPE tissue was peeled away from the sclera, placed in sterile Lysing Matrix D 2.0 mL tubes containing 1.4 mm ceramic spheres (MP Biomedicals), and snap-frozen in liquid nitrogen prior to freezing at −80 °C until analysis. Retinal and choroid-RPE homogenates were analyzed for 8-isoPGF_2α_, H_2_O_2_, SOD, glutathione (GSH), and catalase. All analyses were conducted on the same day. For real-time PCR arrays, retinal and choroid-RPE samples were placed in sterile Lysing Matrix D 2.0 mL tubes containing 1.4 mm ceramic spheres (MP Biomedicals) and snap-frozen in liquid nitrogen prior to freezing at −80 °C until analyses. All analyses were conducted on the same day. For histopathology, whole eyes were fixed in situ in 10% phosphate-buffered formalin, and they were then enucleated and sent to the Pathology Dept. at SUNY Downstate Health Sciences University for processing and embedding using standard techniques. Unstained sections (5 µm) were used for immunoreactivity assays of SOD-1, SOD-2, SOD-3, catalase, HIF_1α_, and VEGF in the retinal layers at *P*21.

### 2.6. Assay of Oxidants

8-isoPGF_2α_ is a reliable and proven biomarker for oxidative stress. H_2_O_2_ is a reactive oxygen byproduct that serves as a key regulator for a number of oxidative stress-related states. Levels in the retina and choroid-RPE homogenates were determined using commercially available ELISA kits (catalogue #s ADI-900-091 and ADI-907-015, respectively) purchased from Enzo Life Sciences (Farmingdale, NY, USA). All samples were processed and assayed according to the manufacturer’s protocols. A total of 6 retina and choroid-RPE samples were analyzed per group from pooled samples within each group. All data were standardized using total cellular protein levels.

### 2.7. Assay of Antioxidants

Levels of SOD, catalase, and total GSH (*n* = 6 samples/group) were determined using commercially available activity assay kits (catalogue #s 706002, 707002, and 703002, respectively) purchased from Cayman Chemical (Ann Arbor, MI, USA). Samples were processed and assayed according to the manufacturer’s protocol and standardized using total cellular protein levels.

### 2.8. Total Cellular Protein Levels

On the day of assays, an aliquot (10 µL) of the retinal and choroid-RPE homogenates was utilized for total cellular protein levels using the Bradford method (catalogue # 5000001, Bio-Rad, Hercules, CA, USA) with bovine serum albumin as a standard.

### 2.9. Immunofluorescence

Unstained cross-sections were deparaffinized with xylenes and alcohol prior to antigen unmasking. Sections were washed several times in PBS containing triton X-100 (PBS-T) and incubated in blocking solution (5% goat serum) for 1 h. The blocking serum was removed, and primary antibodies for SOD-1 (mouse monoclonal IgG, #SC-101523), SOD-2 (mouse monoclonal IgG, #SC-137254), SOD-3 (mouse monoclonal IgG, #SC-271170), catalase (mouse monoclonal IgG, #SC-271803), HIF_1α_ (mouse monoclonal IgG, #SC-13515), and VEGF (mouse monoclonal IgG, #SC-7269) purchased from Santa Cruz Biotechnology (Dallas, TX, USA) were added prior to incubation overnight. The sections were washed and incubated with goat anti-mouse Alexa Fluor fluorescent secondary antibodies (ThermoFisher Sci/Life Technologies, Grand Island, NY, USA) for 1 h. After washing, sections were mounted with DAPI, and images were captured at 20X magnification using an Olympus BX53 microscope, DP72 digital camera, and CellSens imaging software attached to a Dell Precision T3500 computer (Olympus America, Inc., Breinigsville, PA, USA. All buffers were purchased from Boston BioProducts (Ashland, MA, USA). Quantitative analyses were carried out using the count and measure tool of the CellSens software (Olympus America, Center Valley, PA, USA), and their results are presented in Table 1.

### 2.10. Real-Time PCR

Total retinal RNA was extracted as previously described [21,22]. To identify the genes that were affected by neonatal IH, fish oil and CoQ10, real-time PCR arrays were carried out on the retina samples from each group at *P*21 (*n* = 3 samples/group) using the rat mitochondrial PCR array systems (catalogue #PARN-087Z) purchased from Qiagen, Germantown, MD, USA) and a BioRad IQ5 real-time instrument (BioRad, Hercules, CA, USA) per the manufacturer’s instructions. Threshold cycle (C_T_) values were uploaded to the Qiagen GeneGlobe Data Analysis Center for analysis.

### 2.11. Statistical Analysis

Prior to statistical analyses, equality of variances was determined using Bartlett’s test for differences among groups. Data that were normally distributed were analyzed using parametric statistics (two-way analysis of variance, ANOVA) with Dunnett’s multiple comparison post hoc tests, whereas fold changes in genes from RA controls were analyzed using one-way ANOVA with Dunnett’s multiple comparison post hoc tests. Data that were not normally distributed were analyzed using non-parametric statistics (Kruskal Wallis test) with Friedman’s post hoc test. All data are presented as mean ± SEM. A *p*-value of <0.05 (two-tailed) was considered statistically significant. Data were analyzed using SPSS version 16.0 (SPSS Inc., Chicago, IL, USA). Graphs were prepared using GraphPad Prism version 7.03 (GraphPad, San Diego, CA, USA).

## 3. Results

### 3.1. Histopathology

**Figure 1:**
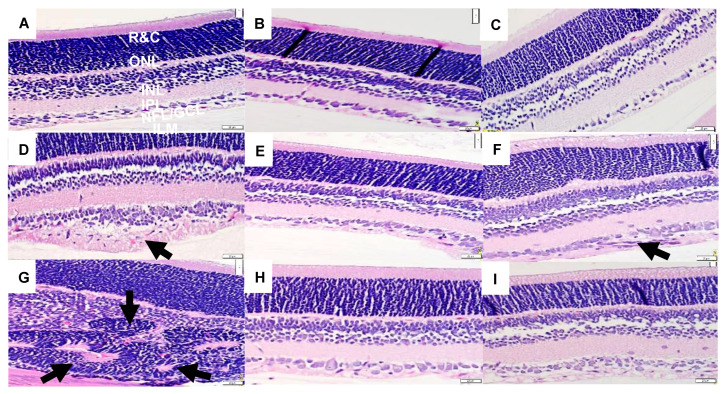
Representative H&E-stained images of retinas from 21-day old rats exposed to room air (RA) or neonatal intermittent hypoxia (IH). Panels (**A**–**C**) represent the RA exposed groups, panels (**D**–**F**) represent the groups exposed to 50/12% O_2_ IH, and panels (**G**–**I**) represent the groups exposed to 21/12% O_2_ IH. Animals supplemented with olive oil (OO) are represented in panels (**A**,**D**,**G**). Animals supplemented with coenzyme Q10 (CoQ10) are represented in panels (**B**,**E**,**H**). Animals supplemented with fish oil are represented in panels (**C**,**F**,**I**). The RA-exposed and OO-supplemented controls show retinal layer integrity with no evidence of pathology. The layers are identified in this group as the following: NFL/GCL (nerve fiber layer/ganglion cell layer); IPL (inner plexiform layer); INL (inner nuclear layer); OPL (outer plexiform layer); ONL (outer nuclear layer); R&C (rods and cones). Images were taken at 40X magnification, and the scale bar is 20 µM. Animals supplemented with OO and exposed to 50/12% O_2_ IH (panel **D**) showed thickening of the NFL/GCL layers and increased numbers of endothelial cells (arrow). Animals supplemented with OO and exposed to 21/12% O_2_ IH (panel **G**) showed hemorrhage and pathological changes in the INL (arrows). Animals treated with CoQ10 and exposed to IH (panels **E** and **H**) showed normalization of the retinal layers, including reduction in the NFL/GCL and number of ECs. Animals treated with fish oil and exposed to IH (panels **F** and **I**) also showed normalization of the retinal layers, although larger vessels were noted in the fish oil group exposed to 50/12% O_2_ IH (arrow).

H&E-stained retinal sections from rats at *P*21 are presented in Figure 1. The retinal layers are identified in the RA olive oil group (panel A), which showed no evidence of retinal pathology. However, the groups supplemented with olive oil and exposed to 50/12% O_2_ IH (panel D) showed increased numbers of retinal endothelial cells (arrow), hemorrhages (arrow), and vitreous condensation, resulting in widening of the nerve fiber layer/ganglion cell layer (NFL/GCL). The groups supplemented with olive oil and exposed to 21/12% O_2_ IH (panel G) showed major distortions in the inner nuclear layer (INL) with the appearance of retinal folds or rosettes (arrow) and hemorrhages (arrow). Supplementation with CoQ10 in 50/12% O_2_ IH (panel E) and 21/12% O_2_ IH (panel H) and fish oil in in 50/12% O_2_ IH (panel F) and 21/12% O_2_ IH (panel I) showed reductions in the number of endothelial cells and narrowing of the NFL/GCL layer; however, the persistence of large vessels (arrow) in the NFL/GCL was evident in the fish oil group exposed to 50/12% O_2_ IH (panel F). Images of further damage in the neonatal IH are presented in Supplementary Figure S1. H&E stains of retinas from untreated groups that were exposed to neonatal IH are presented in Supplemental Figure S2. Images show hemorrhages and major abnormalities.

### 3.2. Oxidative Stress

**Figure 2:**
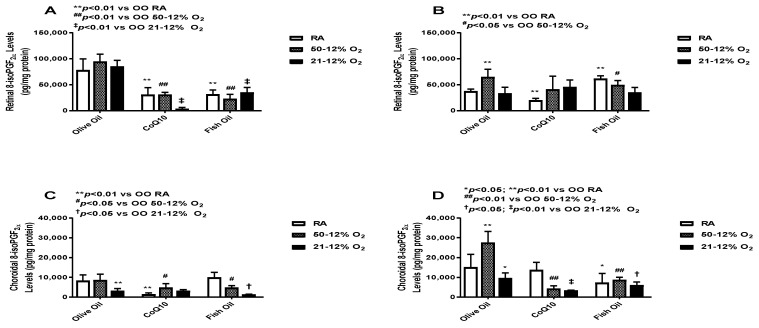
Effects of fish oil or CoQ10 supplementation during neonatal IH on retinal (panels **A** and **B**) and choroid-RPE (panels **C** and **D**) 8-iso-PGF_2α_ levels on postnatal day 14 (panels **A** and **C**) and *P*21 (panels **B** and **D**). Supplementation occurred only during neonatal IH from *P*0 to *P*14. Data are expressed as mean ± SEM. *n* = 6 samples/group.

8-iso-prostaglandin F_2α_ (8-iso-PGF_2α_) is an F_2_-isoprostane, which is a well-established indicator of oxidative stress [23]. The levels of retinal and choroid-RPE 8-iso-PGF_2α_ at *P*14 (panels A and C, respectively) and *P*21 (panels B and D, respectively) are presented in Figure 2. Levels were significantly higher in the OO-treated groups compared to the CoQ10- and fish oil-supplemented groups at *P*14 during exposure. The maximum decrease in the levels of retinal 8-iso-PGF_2α_ was observed in the CoQ10-supplemented 21/12% O_2_ IH group (panel A). At *P*21, this effect was not sustained. At *P*21, the levels of retinal 8-iso-PGF_2α_ in the fish oil-supplemented RA group were high compared to the OO and CoQ10 RA groups (panel B). In choroid-RPE, 8-isoPGF_2α_ levels were lower compared to retinal levels in all groups at *P*14 (panel C). CoQ10 decreased the levels of 8-isoPGF_2α_ in both the RA and 50/12% O_2_ IH groups. Fish oil, on the other hand, led to an increase in 8-isoPGF_2α_ levels in the RA group (panel C). At *P*21, choroid-RPE 8-isoPGF_2α_ levels in the OO-treated groups were higher compared to levels at *P*14. 8-isoPGF_2α_ levels in the CoQ10- and fish oil-supplemented groups were low, especially in the CoQ10-supplemented 50/12% O_2_ IH and 21/12% O_2_ IH groups (panel D).

### 3.3. Hydrogen Peroxide (H_2_O_2_)

**Figure 3:**
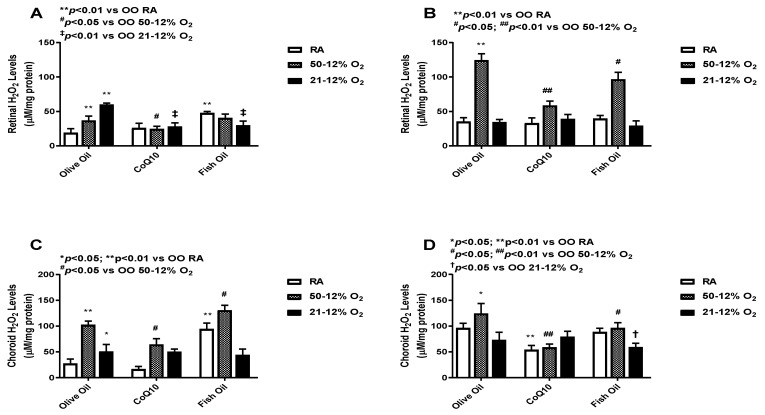
Effects of fish oil or CoQ10 supplementation during neonatal IH on retinal (panels **A** and **B**) and choroid-RPE (panels **C** and **D**) hydrogen peroxide (H_2_O_2_) levels at *P*14 (panels **A** and **C**) and P21 (panels **B** and **D**). Data are expressed as mean ± SEM. *n* = 6 samples/group.

H_2_O_2_ is a marker of oxidative stress, and due to its solubility across membranes, it can cause widespread oxidative damage [24]. Levels in the retina (panels A and B) and choroid-RPE (panels C and D) at *P*14 (panels A and C) and *P*21 (panels B and D) are presented in Figure 3. At *P*14, retinal H_2_O_2_ levels were higher in the OO-supplemented IH groups. CoQ10 supplementation in the IH groups led to a decrease in retinal H_2_O_2_ levels. Fish oil supplementation, on the other hand, led to a decrease in H_2_O_2_ levels only in the 21/12% O_2_ IH group, whereas it led to increased H_2_O_2_ levels in both the RA and 50/12% O_2_ IH groups. Again, we saw an increase in H_2_O_2_ levels in the fish oil-supplemented RA group (panel A). At *P*21, H_2_O_2_ levels were increased in all 50/12% O_2_ IH groups, but less of an increase was seen in the CoQ10-supplemented 50/12% O_2_ IH group compared to both the olive oil- and fish oil-supplemented 50/12% O_2_ IH groups (panel B). At *P*14, choroid-RPE showed higher H_2_O_2_ activity in 50/12 O_2_ IH groups when compared to retinas. The CoQ10-supplemented 50/12% O_2_ IH group showed lower levels of H_2_O_2_ compared to the fish oil-supplemented 50/12% O_2_ IH group. Fish oil supplementation even led to an increase in H_2_O_2_ levels in both the RA and 50/12% O_2_ IH groups (panel C). At *P*21, choroid-RPE H_2_O_2_ levels were higher in all groups, except the fish oil-supplemented 50/12% O_2_ IH group, when compared to *P*14, though less of an increase was seen in the RA and 50/12% O_2_ IH CoQ10-supplemented groups (panel D). The levels of H_2_O_2_ in the retinas and choroid-RPE from untreated animals exposed to neonatal IH are presented in Supplemental Figure S4 in panels A and B, respectively. At *P*14, H_2_O_2_ levels were lower in the 21/12% O_2_ IH group, but these levels increased at *P*21. In the choroid-RPE, levels were higher at *P*14 and *P*21 in the 21/12% O_2_ IH group compared to the 50/12% O_2_ IH group.

### 3.4. Superoxide Dismutase (SOD)

**Figure 4:**
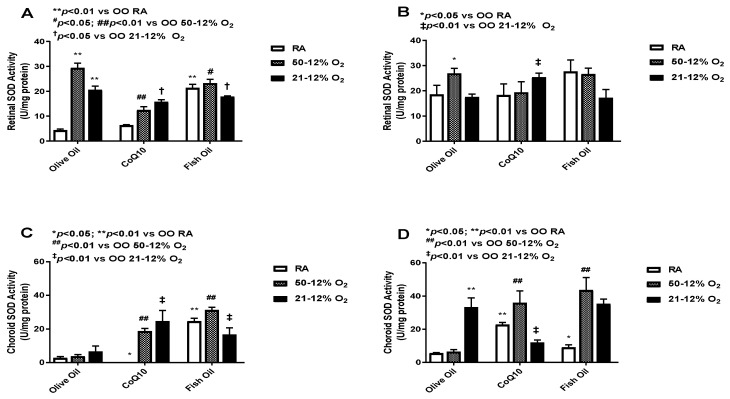
Effects of fish oil or CoQ10 supplementation during neonatal IH on retinal (panels **A** and **B**) and choroid-RPE (panels **C** and **D**) superoxide dismutase (SOD) levels on *P*14 (panels **A** and **C**) and *P*21 (panels **B** and **D**). Data are expressed as mean ± SEM. *n* = 6 samples/group.

SOD dismutates superoxide anions and converts them into H_2_O_2_ and O_2_ [25]. SOD levels in the retinas (panels A and B) and choroid-RPE (panels C and D) at *P*14 (panels A and C) and *P*21 (panels B and D) are presented in Figure 4. At *P*14, retinal SOD activity was high in both IH groups. Retinal SOD activity was also high in the RA group but only in rat pups who had received fish oil supplementation. CoQ10 supplementation decreased retinal SOD activity in both IH groups. Fish oil supplementation also led to reductions in retinal SOD activity in both IH groups, but the effect was lower compared to the effect seen with CoQ10. SOD activity increased in the fish oil-supplemented RA group (panel A). At *P*21, retinal SOD levels were increased compared to SOD levels at *P*14; this effect was noticed across all groups, which means that the effect of the treatment was not sustained. The CoQ10-supplemented 50/12% O_2_ IH group had lower SOD levels when compared to the fish oil- and olive oil-supplemented 50/12% O_2_ IH groups. Again, fish oil showed an increase in SOD activity in RA group at *P*21 (panel B). At *P*14, SOD activity levels in the choroid-RPE was low compared to retinal SOD activity. The CoQ10-supplemented 50/12% O_2_ IH group showed a lower level of choroid-RPE SOD activity compared to the fish oil-supplemented group. There was an increase in SOD levels in the fish oil-supplemented RA group (panel C). Choroid-RPE SOD activity at *P*21 was overall increased in all groups, except the CoQ10-supplemented 21/12% O_2_ IH and fish oil-supplemented RA groups, when compared to *P*14. SOD activity was higher in both of the IH fish oil-supplemented groups compared to the CoQ10 group (panel D). The levels of SOD in the retinas and choroid-RPE from untreated animals exposed to neonatal IH are presented in Supplementary Figure S4 in panels C and D, respectively. In retinas, SOD was increased for the 21/12% O_2_ IH group at *P*21, whereas in choroid-RPE, SOD was increased at *P*14 and *P*21, coincident with H_2_O_2_ levels, in the same group.

### 3.5. Catalase

**Figure 5:**
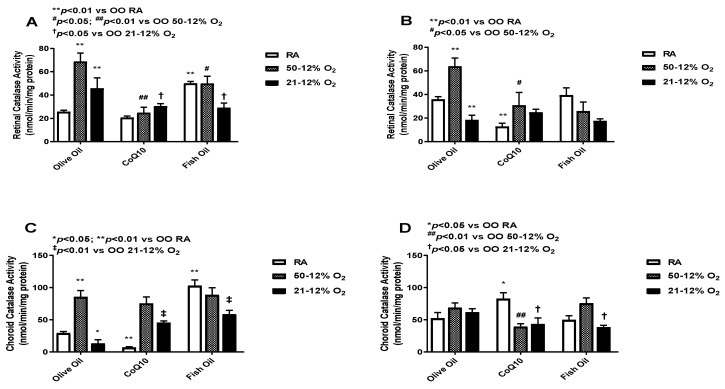
Effects of fish oil or CoQ10 supplementation during neonatal IH on retinal (panels **A** and **B**) and choroid-RPE (panels **C** and **D**) catalase levels on *P*14 (panels **A** and **C**) and *P*21 (panels **B** and **D**). Data are expressed as mean ± SEM. *n* = 6 samples/group.

Catalase converts H_2_O_2_ into water and O_2_. Catalase works more efficiently when H_2_O_2_ levels are high [26]. The levels of catalase in the retinal (panels A and B) and choroid-RPE (panels C and D) homogenates are presented in Figure 5. At *P*14, retinal catalase activity was higher in the olive oil-supplemented IH groups compared to the olive oil RA group. The CoQ10- and fish oil-supplemented groups had much lower levels of retinal catalase in IH compared to the olive oil IH groups. At *P*21, retinal catalase activity remained low in the CoQ10- and fish oil-supplemented groups compared to olive oil in the 50/12% O_2_ group (panel B). Given the role of catalase in H_2_O_2_ detoxification, these findings may suggest a reduction in lipid peroxidation. At *P*14, the CoQ10-supplemented RA group showed the lowest choroid-RPE catalase activity compared to the fish oil RA group, which showed the highest catalase activity (panel C). At *P*21, the highest catalase activity in choroid-RPE was seen in the CoQ10 RA group, which was minimal at *P*14. Choroid-RPE catalase levels were low in both the CoQ10 and fish oil 21/12% O_2_ IH groups (panel D). Levels of catalase in the retinas and choroid-RPE from untreated animals exposed to neonatal IH are presented in Supplemental Figure S5 in panels A and B, respectively. In retinas, catalase was increased in the 21/12% O_2_ IH groups at *P*14 and *P*21, whereas in the choroid-RPE, catalase was increased at *P*14.

### 3.6. Glutathione

**Figure 6:**
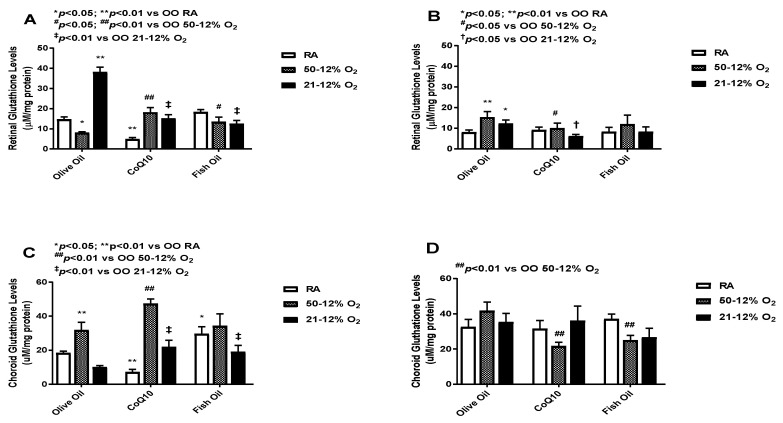
Effects of fish oil or CoQ10 supplementation during neonatal IH on retinal (panels **A** and **B**) and choroid-RPE (panels **C** and **D**) glutathione (GSH) levels on *P*14 (panels **A** and **C**) and *P*21 (panels **B** and **D**). Data are expressed as mean ± SEM. *n* = 6 samples/group.

Glutathione (GSH) acts as a scavenger of free radicals [27,28]. The levels of total GSH in the retinal (panels A and B) and choroid-RPE (panels C and D) homogenates are presented in Figure 6. At *P*14, CoQ10 and fish oil supplementation led to an increase in glutathione levels in the 50/12% O_2_ IH groups. Further increases were seen with CoQ10 supplementation. The highest GSH level was found in the olive oil 21/12% O_2_ IH group, and CoQ10 and fish oil supplementation led to declines in its level (panel A). At *P*21, higher levels of retinal GSH were observed in the OO 50/12% O_2_ IH and 21/12% O_2_ IH groups compared to the OO RA group. The effect of treatment in retinas was not sustained at *P*21, as evidenced by declining GSH levels (panel B). Compared to those in retinas, the choroid-RPE levels of GSH were higher in all groups at *P*14 (panel C) and *P*21 (panel D). At *P*14, the maximum increased level of GSH was seen in the 50/12% O_2_ IH CoQ10-supplemented group. CoQ10 and fish oil both increased the GSH levels in the 21/12% O_2_ IH groups compared to the OO 21/12% O_2_ IH group. At *P*21, the effect of treatment was more sustained in choroid-RPE compared to retinas. Levels of glutathione in the retinas and choroid-RPE from untreated animals exposed to neonatal IH are presented in Supplementary Figure S5 in panels C and D, respectively. In retinas, glutathione was increased in the 21/12% O_2_ IH group at *P*21, whereas in choroid-RPE, glutathione was increased at *P*14.

### 3.7. Immunofluorescence

**Figure 7:**
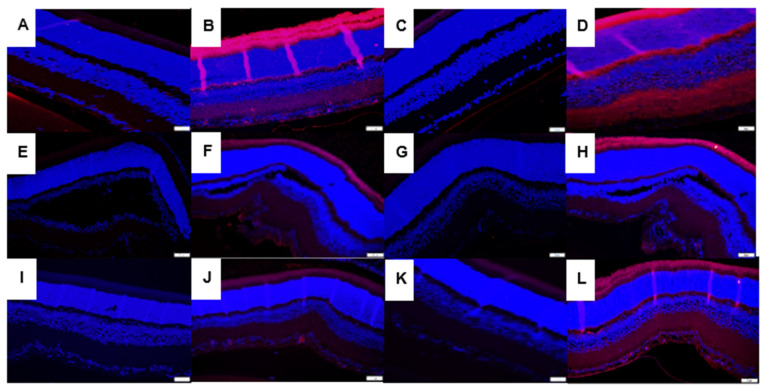
Representative images showing the immunoreactivity of SOD-3 (panels **A**,**B**,**E**,**F**,**I**,**J**) and catalase (panels **C**,**D**,**G**,**H**,**K**,**L**) in the retinal sections from groups supplemented with CoQ10 in RA (panels A and C, respectively), 50/12% O_2_ IH (panels **E** and **G**, respectively), and 21/12% O_2_ IH (panels **I** and **K**, respectively) as well as those supplemented with fish oil in RA (panels **B** and **D**, respectively), 50/12% O_2_ IH (panels **F** and **H**, respectively), and 21/12% O_2_ IH (panels **I** and **K**, respectively). CoQ10 decreased SOD-3 in RA (panel **A**), 50/12% O_2_ IH (panel **E**), and 21/12% O_2_ IH (panel **I**), and it decreased catalase in RA (panel **C**), 50/12% O_2_ IH (panel **G**), and 21/12% O_2_ IH (panel **K**) compared to fish oil. The corresponding quantitative analysis results are presented in Table 1. Images were taken at 40× magnification (scale = 20 µM).

Immunoreactivities of SOD-3 (extracellular) and catalase in retinas at *P*21 are presented in Figure 7. Panels A–D represent the RA groups, panels E–H represent the 50/12% O_2_ IH groups, and panels I–J represent the 21/12% O_2_ IH groups. Panels A, E, and I represent SOD-3 immunoresponses in the CoQ10-supplemented groups exposed to RA (panel A), 50/12% O_2_ IH (panel E), and 21/12% O_2_ (panel I), respectively. Panels B, F, and J represent SOD-3 immunoresponses in the fish oil-supplemented groups exposed to RA (panel A), 50/12% O_2_ IH (panel E), and 21/12% O_2_ (panel I), respectively. Panels C, G, and K represent catalase immunoresponses in the CoQ10-supplemented groups exposed to RA (panel C), 50/12% O_2_ IH (panel G), and 21/12% O_2_ (panel K), respectively. Panels D, H, and L represent catalase immunoresponses in the fish oil-supplemented groups exposed to RA (panel D), 50/12% O_2_ IH (panel H), and 21/12% O_2_ (panel L), respectively. Both fish oil and CoQ10 had no substantive effect on SOD-1 or SOD-2 (data not shown). In contrast, SOD-3 was completely suppressed with CoQ10 in all retinal layers and in all oxygen environments, an effect that was less potent with fish oil. Similar findings were noted for catalase immunoreactivity. Analysis of HIF_1α_ and VEGF immunoreactivities (Figure S3) showed that both fish oil and CoQ10 suppressed HIF_1α_ in the NFL/GCL layer, but the suppressive effect was more substantial with CoQ10. Although the expression of VEGF was almost abolished with CoQ10, fish oil caused substantial increases in VEGF expression in the NFL/GCL, inner plexiform layer (IPL), rods and cones, and choroid-RPE, particularly in the 50/12% O_2_ IH group, and it was consistent with higher H_2_O_2_ (shown in Figure 3) and SOD (shown in Figure 4) levels in the choroid-RPE. The corresponding quantitative analysis results for all IF images are presented in Table 1.

### 3.8. Mitochondrial RT^2^ Profiler PCR Array in the Retina

Mitochondrial gene expression profile in the retina is presented in Table 2. Only genes that were expressed >10-fold compared to RA controls are presented; those presented are organized according to their function. Of the 88 genes in the PCR array panel, we found that both IH paradigms significantly upregulated genes involved in mitochondrial transport and in outer (*Tomm*) and inner (*Timm*) membrane translocation (13-fold to 35-fold, *p* < 0.05) compared to RA controls. Membrane translocator genes also help with the functioning and formation of SOD-2. Supplementation with both CoQ10 (28-fold, *p* < 0.05) and fish oil (21-fold, *p* < 0.05) in 21/12% O_2_ IH upregulated those genes. We also found that glutamate-cysteine ligase catalytic gene (*Gclc*) and glutamate-cysteine ligase modifier gene (*Gclm*) expression levels, which help in the formation of GSH and maintain mitochondrial membrane polarization and potential, were lower with IH (2-fold and 4-fold, respectively) and fish oil (3-fold and 8-fold, respectively), but they were higher with CoQ10 (14-fold and 9-fold, respectively), although significance was not achieved. The same response was seen with SOD-1 gene expression. Regarding uncoupling proteins (*Ucp*), which are important for maintaining body temperature and energy balance and protecting against oxidative stress, these were also expressed more in CoQ10-supplemented IH groups (up to 10-fold) compared to OO and fish oil, in which the expression levels were downregulated with fish oil (2-fold). Solute carrier family 25 (*Slc25*) genes, and *Slc25a3* in particular, which encodes transporters in the inner mitochondrial membrane, were also highly expressed with CoQ10 (58-fold, *p* < 0.05) in RA, 43-fold (*p* < 0.05) in 50/12% O_2_ IH, and 42-fold (*p* < 0.05) in 21/12% O_2_ IH. Although treatment with OO and fish oil also increased *Slc25a3* compared to RA OO controls, their expression levels were lower than with CoQ10. Overall, CoQ10 supplementation increased all *Slc25* genes to a greater extent than fish oil.

## 4. Discussion

The effects of CoQ10 and fish oil on weight accretion, eye opening, and angiogenic factors are presented elsewhere [18,20]. The major findings of this study were reduced ocular oxidative stress and improved retinal outcomes with CoQ10. This was evidenced by the levels of H_2_O_2_ and 8-iso-PGF_2α_ in treated animals. In addition, the beneficial effects of CoQ10 were associated with the complete abrogation of SOD-3, catalase, HIF_1α_ and VEGF in the inner retina as well as upregulation of the retinal *Gclc*, *Gclm*, *Ucp*, and *Slc25* genes. This is the first study to demonstrate these associations in response to CoQ10. In contrast, although fish oil did indeed reduce oxidative stress biomarkers and their resulting reductions in antioxidants, the effect was less potent than that of CoQ10. Surprisingly, we noted increased expression levels of HIF_1α_ and VEGF with fish oil. CoQ10 supplementation during neonatal IH decreases ROS build-up by inducing antioxidants. Although fish oil supplementation showed beneficial effects on growth [18], protection against IH-induced OIR was inadequate [20,29]. These findings imply a potential therapeutic role for CoQ10 in ELBW neonates at risk for “oxygen radical diseases in neonatology (ORDIN)” [16,30].

Mitochondria are essential components of cells. They participate in intracellular signaling and cellular metabolism. The primary role of mitochondria is the generation of adenosine triphosphate (ATP) through oxidative phosphorylation (OXPHOS) via five enzyme complexes [31]. ROS buildup due to immature antioxidant systems can significantly impact mitochondrial function. Indeed, we noted that both IH paradigms caused retinal degeneration. In particular, retinas exposed to 21/12% O_2_ IH showed severe abnormalities and punctate hemorrhages in the NFL/GCL, INL, and ONL, which contain high amounts of mitochondria. These adverse outcomes appear to be related to H_2_O_2_ accumulation, and they were reversed with CoQ10. Superoxide anions and H_2_O_2_ trigger apoptosis and cell death [32]. The stability and membrane-permeability of H_2_O_2_ is likely to cause widespread cell damage. The retina also has high oxygen demands because of its high mitochondria content, which is necessary for photo-transduction and energy production [33]. Indeed, we showed significant damage to the INL with severe hemorrhages (Figure 1, panel G). Whether this damage is due to hemorrhage remains to be determined. However, early studies by Ashton et al. [34] showed similar abnormalities in the retinas of kittens exposed to hyperoxia. Other animal studies also showed distorted INL and ONL [35,36]. These previous studies suggest that these abnormalities were indicative of severe photoreceptor death, retinal gliosis, and retinal detachment. Based on our current findings, we propose that neonatal IH experienced by ELGANs alters the balance of ROS and antioxidants and disturbs the redox homeostasis of the retina, leading to accumulation of H_2_O_2_ and, subsequently, lipid peroxidation and retinal damage, which may be curtailed with CoQ10. CoQ10’s fat solubility protects against lipid peroxidation in tissues with high lipid content, such as those in the eyes. Its elimination half-life is about 33 h, as demonstrated in adult studies [37]. CoQ10’s absorption increases if it is combined and ingested with a high fat diet [38]. The benefits of CoQ10 for retinal diseases were previously demonstrated [39].

SOD detoxifies superoxide anions, the primary ROS produced in the body. SOD plays a central role in providing defense against oxidative stress. The levels of SOD increase in response to superoxide anions and are indicative of increased oxidative stress [40]. Our study shows that in the retina, CoQ10 was superior to fish oil for reducing SOD during neonatal IH, which suggests a reduction in oxidative stress and is consistent with lower H_2_O_2_ levels. In a previous study, we showed that high doses of the SOD mimetic, MnTBAP, led to worsening of oxygen-induced retinopathy, whereas lower doses were preventative [41]. This suggests that high doses of exogenous SOD can result in the accumulation of H_2_O_2_ due to inefficient scavenging by catalase and/or glutathione. In another study, it was found that recombinant human Cu/Zn superoxide dismutase (rhSOD) reduced the risk of developing ROP in ELGANs. ROP was reduced by 53% from 85% (placebo) to 40% (rhSOD) (*p* = 0.03) in babies born at <25 weeks of gestation [42]. It is clear that a redox balance is important. H_2_O_2_’s reaction with hydroxyl radicals via the Haber–Weiss reaction readily attacks the lipid membrane in the retina, resulting in a self-perpetuating chain reaction [11] that may be the major culprit in the development of severe ROP.

Catalase is mainly found in peroxisomes and cytoplasm, where its main function is to detoxify H_2_O_2_, which is a major redox metabolite involved in redox sensing, signaling, and regulation. Although both CoQ10 and fish oil decreased catalase in retinas and choroid-RPE, only CoQ10 successfully reduced H_2_O_2_. Catalase is often co-distributed with SOD, but it is more efficient when H_2_O_2_ is at toxic levels [43]. Our finding suggests that H_2_O_2_ levels were not high enough to induce a catalase response. In this regard, GSH may play a more important role for maintaining the redox balance and the proper functioning of the cells [44,45]. Accumulation of fatty acids in cells may perpetuate lipid peroxidation reactions. A study examining the glutathione status of red blood cells in infants with ROP showed low levels of reduced and high levels of oxidized GSH, suggesting a redox imbalance [46]. In a mouse model of ROP, glutathione peroxidase 1 knockout resulted in increased oxidative stress, upregulation of retinal VEGF, and worse retinal outcomes [47]. In our study, retinal GSH was highly increased in response to 21/12% O_2_ IH in the group treated with OO and was coincident with high H_2_O_2_ levels. This confirms manipulation via GSH and not catalase. Choroid-RPE levels of H_2_O_2_ were higher than retinal levels, suggesting more accumulation of H_2_O_2_ there, as we previously showed [22].

## 5. Conclusions

The current findings improve on our previous work using a single IH paradigm with recovery in hyperoxia between each IH episode, which shows that CoQ10 and fish oil did not completely prevent severe OIR. In the current study, we also used an IH paradigm with recovery in normoxia between episodes to determine whether hyperoxia was responsible for the diminished efficacy, considering that both CoQ10 and fish oil have antioxidant properties which may decline in hyperoxia. We demonstrated that exposure to IH, regardless of recovery in hyperoxia or RA between episodes, caused severe retinal abnormalities. These abnormalities were more severe in the group exposed to 21/12% O_2_ IH. This may be primarily due to a hypoxia effect, whereas in the 50/12% O_2_ IH group, hyperoxia may offset the mechanisms induced by hypoxia. We also show that both IH paradigms are indeed associated with oxidative stress and impairment of retinal antioxidant status. Finally, we show that early supplementation with fish oil or CoQ10 is beneficial, but CoQ10 is by far superior to fish oil for reducing IH-induced retinal oxidative stress and injury. The etiology of OIR is multifactorial. In this regard, there is no single therapy that targets all the key factors involved in its development and progression. However, both supplements demonstrated individual attributes. Whether combining the treatments would result in synergistic beneficial outcomes remains to be determined. Although the nutritional benefits of fish oil for improving growth is clear, its therapeutic effects for reducing IH-induced OIR remain suboptimal compared to CoQ10, which may be due to an accumulation of lipids which are targets for lipid peroxidation. Although CoQ10 was proven to be safe in this animal model, its use in preterm infants requires further dose-finding and safety studies.

## Figures and Tables

**Table 1:** Quantitative Analysis of Immunoreactivity.

	HIF_1α_	VEGF	SOD-1	SOD-2	SOD-3	Catalase
RA:						
Olive Oil	529.8 ± 140.2	907.3 ± 199.0	949.6 ± 95.4	619.9 ± 64.4	674.3 ± 147.6	715.6 ± 91.2
CoQ10	133.1 ± 13.3 *	869.2 ± 121.2	989.5 ± 90.8	919.2 ± 147.9	468.1 ± 99.7	283.8 ± 51.2 **
Fish Oil	1029.3 ± 108.6 **	1958.0 ± 153.9 **	1599.7 ± 155.5 **	661.0 ± 123.9	945.0 ± 54.9	852.7 ± 123.8
50/12% IH:						
Olive Oil	1399.1 ± 230.1 ^##^	1150.1 ± 164.6	1297.5 ± 45.5 ^#^	1257.0 ± 191.9 ^##^	1337.7 ± 134.9 ^##^	1997.2 ± 173.1^##^
CoQ10	276.6 ± 51.9 **^#^	471.5 ± 46.1 **^##^	1242.3 ± 86.9	712.6 ± 56.1 *	115.6 ± 18.7 **^##^	160.6 ± 15.5 **^#^
Fish Oil	1329.6 ± 219.0	1445.8 ± 112.1 ^##^	985.0 ± 110.4 *^##^	874.4 ± 126.8	770.0 ± 88.5 **	650.3 ± 125.9 **
21/12% IH:						
Olive Oil	1145.8 ± 36.8 ^#^	1456.4 ± 96.4	1475.8 ± 128.2 ^##^	1308.8 ± 63.2 ^##^	970.3 ± 66.2	830.3 ± 122.8
CoQ10	257.1 ± 28.5 **^#^	457.7 ± 47.9 **^##^	1276.6 ± 75.4 ^#^	1144.2 ± 124.2	204.6 ± 14.9 **^##^	150.2 ± 12.5 **^#^
Fish Oil	869.1 ± 84.0 **	1077.4 ± 83.5 **^##^	1120.1 ± 81.8 *^#^	1034.8 ± 79.1 ^#^	576.0 ± 54.8 **^##^	877.6 ± 137.9

Data were analyzed using two-way ANOVA with Dunnett’s post hoc test. Data are mean ± SEM (*n* = 12 measurements/group). * *p* < 0.05, ** *p* < 0.01 vs. olive oil; ^#^ *p* < 0.05, ^##^ *p* < 0.01 vs room air (RA). HIF (hypoxia inducible factor); VEGF (vascular endothelial growth factor); SOD (superoxide dismutase); IH (intermittent hypoxia).

**Table 2:** Fold Change in Expression of Mitochondrial Genes Compared to Olive Oil Room Air Controls.

Genes of Interest	CoQ10 RA	Fish Oil RA	Olive Oil 50/12%	CoQ10 50/12%	Fish Oil 50/12%	Olive Oil 21/12%	CoQ10 21/12%	Fish Oil 21/12%
Membrane Polarization and Potential:								
*Bnip3*	−1.4 ± 0.09	23.9 ± 0.2	11.9 ± 0.2	17.8 ± 0.06	15.4 ± 0.16	21.9 ± 0.64	32.9 ± 0.68	20.1 ± 0.18
*Gclc*	10.1 ± 0.36	4.7 ± 0.56	2.4 ± 0.37	14.1 ± 0.68	3.3 ± 0.53	4.4 ± 0.82	8.5 ± 0.48	3.9 ± 0.59
*Gclm*	9.6 ± 0.35	4.6 ± 0.35	3.4 ± 0.3	7.8 ± 0.34	5.7 ± 0.33	7.9 ± 0.39	13.2 ± 0.35	8.0 ± 0.3
*Sod1*	10.7 ± 0.32	2.3 ± 0.36	4.1 ± 0.27	9.6 ± 0.34	8.3 ± 0.39	6.0 ± 0.28	11.9 ± 0.31	7.9 ± 0.3
Mitochondrial Transport and Targeting Proteins to Mitochondria:								
*Grpel1*	−1.4 ± 0.17	11.5 ± 0.24	8.6 ± 0.46	10.0 ± 0.22	9.2 ± 0.31	12.6 ± 0.46	17.7 ± 0.33	10.0 ± 0.24
*Hsp90aa1*	2.9 ± 0.03 *	11.3 ± 0.08	5.3 ± 0.05 *	8.7 ± 0.04 *	9.8 ± 0.09	9.8 ± 0.08	15.5 ± 0.07	11.0 ± 0.08
*Hspd1*	−1.6 ± 0.28	38.2 ± 0.95	11.8 ± 0.31	21.9 ± 0.35	26.7 ± 0.71	29.2 ± 0.77	46.6 ± 0.83	6.5 ± 0.05 *
*Mfn2*	7.0 ± 0.53	11.1 ± 0.4	5.6 ± 0.41	9.5 ± 0.39	8.7 ± 0.44	9.9 ± 0.46	14.3 ± 0.42	8.9 ± 0.38
*Mipep*	4.0 ± 39	8.3 ± 0.39	5.1 ± 0.41	6.2 ± 0.36	5.2 ± 0.37	7.4 ± 0.41	10.3 ± 0.38	5.7 ± 0.35
*Mtx2*	−1.2 ± 18	13.6 ± 0.29	6.7 ± 0.31	12.3 ± 0.42	11.1 ± 0.28	9.7 ± 0.42	17.9 ± 0.33	11.1 ± 0.27
*Timm10*	12.2 ± 0.85	6.4 ± 0.78	13.7 ± 0.02 *	21.0 ± 0.36	18.2 ± 0.24	13.1 ± 0.01 *	26.3 ± 0.11	19.5 ± 0.42
*Ucp1*	11.2 ± 0.37	−1.8 ± 0.3	−1.1 ± 0.7	4.9 ± 0.4	−1.7 ± 0.59	4.8 ± 0.93	10.6 ± 0.25	−1.8 ± 0.36
*Ucp2*	3.6 ± 0.42	4.4 ± 0.37	2.7 ± 0.38	4.9 ± 0.73	3.4 ± 0.63	2.7 ± 0.43	8.9 ± 0.19	2.2 ± 0.49
*Ucp3*	−1.9 ± 0.35	−1.1 ± 0.38	1.8 ± 0.27	2.2 ± 0.46	−1.8 ± 0.33	4.7 ± 0.19	2.3 ± 0.38	−2.0 ± 0.42
Small Molecule Transport:								
*Slc25a12*	8.4 ± 0.62	23.9 ± 0.8	8.9 ± 0.57	13.2 ± 0.49	12.1 ± 0.59	12.1 ± 0.54	20.1 ± 0.54	10.1 ± 0.41
*Slc25a14*	2.6 ± 0.28	10.5 ± 0.36	5.5 ± 0.37	8.8 ± 0.35	8.4 ± 0.41	8.8 ± 0.4	12.3 ± 0.35	9.2 ± 0.36
*Slc25a16*	3.2 ± 0.3	12.3 ± 0.41	5.3 ± 0.36	9.2 ± 0.39	8.0 ± 0.38	8.5 ± 0.37	13.6 ± 0.35	8.8 ± 0.28
*Slc25a17*	1.5 ± 0.24	14.5 ± 0.42	7.3 ± 0.43	11.1 ± 0.36	11.3 ± 0.52	10.4 ± 0.41	16.6 ± 0.39	12.9 ± 0.43
*Slc25a22*	6.8 ± 0.55	12.0 ± 0.44	7.0 ± 0.48	9.7 ± 0.44	7.6 ± 0.43	8.1 ± 0.38	13.1 ± 0.4	5.6 ± 0.56
*Slc25a23*	10.7 ± 0.89	21.8 ± 0.67	17.8 ± 0.05 *	21.4 ± 0.86	22.6 ± 0.09	22.9 ± 0.2	34.7 ± 0.43	15.8 ± 0.16
*Slc25a27*	4.3 ± 0.33	11.3 ± 0.37	6.7 ± 0.41	7.7 ± 0.3	8.1 ± 0.38	8.5 ± 0.36	13.9 ± 0.36	4.5 ± 0.39
*Slc25a3*	58.2 ± 0.01 *	15.0 ± 0.01 *	24.7 ± 0.05 *	43.4 ± 0.06 *	30.6 ± 0.01 *	27.4 ± 0.04 *	41.7 ± 0.05 *	6.1 ± 0.02 *
*Slc25a36*	1.2 ± 0.22	18.2 ± 0.57	8.2 ± 0.53	9.2 ± 0.37	10.0 ± 0.49	7.2 ± 0.37	14.2 ± 0.39	11.6 ± 0.39
*Slc25a4*	3.1 ± 0.3	28.7 ± 0.05 *	14.5 ± 0.01 *	23.3 ± 0.03 *	20.9 ± 0.01 *	16.2 ± 0.01 *	34.7 ± 0.01 *	25.0 ± 0.01 *
*Slc25a5*	6.3 ± 0.83	20.5 ± 0.6	12.4 ± 0.97	18.6 ± 0.69	14.9 ± 0.77	13.2 ± 0.47	33.0 ± 0.84	18.2 ± 0.68
Mitochondrial Protein Import:								
*Cav2*	−4.4 ± 0.16	12.1 ± 0.3	5.4 ± 0.26	9.7 ± 0.41	10.2 ± 0.31	9.2 ± 0.24	12.1 ± 0.64	8.1 ± 0.22
*Gpx1*	11.3 ± 0.8	13.2 ± 0.7	9.3 ± 0.12	20.5 ± 0.01 *	11.5 ± 0.05 *	16.0 ± 0.35	17.8 ± 0.03 *	14.0 ± 0.05 *
*Grpel1*	−1.4 ± 0.17	11.5 ± 0.24	8.6 ± 0.46	10.0 ± 0.23	9.2 ± 0.31	12.6 ± 0.46	17.7 ± 0.33	10.0 ± 0.24
*Hspd1*	−1.6 ± 0.28	38.2 ± 0.05 *	11.8 ± ±0.31	21.9 ± 0.35	26.7 ± 0.71	29.2 ± 0.03 *	46.6 ± 0.03 *	6.5 ± 0.53
*Mipep*	4.0 ± 39	8.3 ± 0.39	5.1 ± 0.41	6.2 ± 0.36	5.2 ± 0.37	7.4 ± 0.41	10.3 ± 0.38	5.7 ± 0.35
*Ppargc1a*	4.0 ± 0.19	10.6 ± 0.23	5.0 ± 0.19	10.2 ± 0.23	11.1 ± 0.42	8.7 ± 0.23	13.6 ± 0.22	9.7 ± 0.22
*Sh3glb1*	12.5 ± 0.64	29.0 ± 0.01 *	11.5 ± 0.01 *	23.2 ± 0.65	21.6 ± 0.01 *	19.4 ± 0.57	32.0 ± 0.8	21.3 ± 0.12
*Timm10*	12.2 ± 0.85	6.4 ± 0.78	13.7 ± 0.02 *	21.0 ± 0.36	18.2 ± 0.24	13.1 ± 0.01 *	26.3 ± 0.11	19.5 ± 0.42
Outer Membrane Translocation								
*Tomm22*	6.0 ± 0.38	4.4 ± 0.37	7.0 ± 0.53	12.3 ± 0.61	11.3 ± 0.49	12.6 ± 0.5	16.7 ± 0.36	12.3 ± 0.38
*Tomm34*	4.0 ± 0.34	19.6 ± 0.38	14.8 ± 0.03 *	3.7 ± 0.4	7.0 ± 0.41	18.0 ± 0.03 *	8.1 ± 0.36	7.2 ± 0.5
*Tomm40*	3.0 ± 0.39	6.0 ± 0.37	5.5 ± 0.44	6.8 ± 0.4	4.0 ± 0.38	11.1 ± 0.41	9.5 ± 0.4	3.9 ± 0.36
*Tomm70*	6.4 ± 0.34	17.4 ± 0.47	9.1 ± 0.5	13.5 ± 0.36	11.9 ± 0.48	11.6 ± 0.47	16.4 ± 0.28	11.3 ± 0.38
Inner Membrane Translocation:								
*Immp1l*	1.9 ± 0.17	16.7 ± 0.43	5.9 ± 0.22	12.0 ± 0.29	12.0 ± 0.47	5.0 ± 0.11	17.1 ± 0.29	7.9 ± 0.16
*Opa1*	1.8 ± 0.07	26.8 ± 0.58	12.5 ± 0.01 *	21.1 ± 0.01 *	19.3 ± 0.01 *	21.3 ± 0.01 *	30.4 ± 0.01 *	28.2 ± 0.01 *
*Timm10*	12.2 ± 0.85	6.4 ± 0.78	13.7 ± 0.02 *	21.0 ± 0.36	18.2 ± 0.24	13.1 ± 0.01 *	26.3 ± 0.11	19.5 ± 0.42
*Timm17a*	6.0 ± 0.46	11.3 ± 0.38	6.2 ± 0.42	8.9 ± 0.36	7.5 ± 0.39	8.1 ± 0.38	10.7 ± 0.33	7.5 ± 0.34
*Timm22*	6.4 ± 0.53	13.8 ± 0.43	6.6 ± 0.43	10.6 ± 0.4	8.8 ± 0.42	9.0 ± 0.4	14.8 ± 0.51	9.4 ± 0.38
*Timm44*	3.3 ± 0.33	10.1 ± 0.45	6.5 ± 0.42	9.2 ± 0.39	6.1 ± 0.36	9.8 ± 0.43	11.1 ± 0.36	8.6 ± 0.38
*Timm8a1*	8.1 ± 0.41	18.1 ± 0.25	8.2 ± 0.17	17.2 ± 0.36	12.7 ± 0.32	10.6 ± 0.11	21.2 ± 0.22	14.8 ± 0.31
*Timm8b*	2.2 ± 0.56	15.2 ± 0.75	35.2 ± 0.05 *	14.7 ± 0.86	10.8 ± 0.63	20.0 ± 0.04 *	14.8 ± 0.66	12.8 ± 0.48
*Timm9*	3.0 ± 0.21	13.4 ± 0.34	5.1 ± 0.27	11.7 ± 0.36	8.8 ± 0.53	6.7 ± 0.49	13.1 ± 0.36	7.0 ± 0.38
Apoptosis:								
*Bnip3*	−1.4 ± 0.09	23.9 ± 0.2	11.9 ± 0.2	17.8 ± 0.06	15.4 ± 0.16	21.9 ± 0.64	32.9 ± 0.68	20.1 ± 0.18
*Dnm1l*	4.7 ± 0.16	19.6 ± 0.45	10.1 ± 0.49	18.8 ± 0.36	16.1 ± 0.35	14.5 ± 0.4	21.7 ± 0.32	16.2 ± 0.29
*Gpx1*	11.3 ± 0.8	13.2 ± 0.07 *	9.3 ± 0.12	20.5 ± 0.06 *	11.5 ± 0.7	16.0 ± 0.35	17.9 ± 0.03 *	14.0 ± 0.05 *
*Sh3glb1*	12.5 ± 0.64	29.0 ± 0.01 *	11.5 ± 0.01 *	23.2 ± 0.65	21.6 ± 0.01 *	19.4 ± 0.57	32.0 ± 0.8	21.3 ± 0.12
*Sod2*	11.4 ± 0.64	14.5 ± 0.48	25.2 ± 0.07	22.5 ± 0.09	26.9 ± 0.07	30.9 ± 0.01	20.6 ± 0.06 *	28.4 ± 0.03 *
*P53*	4.6 ± 0.46	9.8 ± 0.4	4.4 ± 0.39	6.2 ± 0.37	6.7 ± 0.39	6.0 ± 0.38	10.1 ± 0.53	6.6 ± 0.75

Of the 88 genes in the mitochondrial array, only those genes with >10-fold expression in at least one group are presented. Data were analyzed using one-way ANOVA with Dunnett’s post hoc test. Data are represented as mean ± SEM (three replicates/group). * *p* < 0.05 vs. Olive Oil RA. Genes of interest in alphabetical order: *Bnip3* (BCL2-interacting protein 3); *Cav2* (caveolin 2); *Dnm1l* (dynamin-like protein); *Gclc* (glutamate cysteine ligase catalytic); *Gclm* (glutamate cysteine ligase modifier); *Gpx* (glutathione peroxidase); *Grpel1* (GrpE-like 1); *Hsp90aa1* (heat shock protein 90); *Hspd1* (heat shock protein 1); *Immp1l* (IMP1 inner mitochondrial membrane peptidase-like); *Mfn2* (mitofusion 2); *Mipep* (mitochondrial intermediate peptidase); *Mtx2* (Metaxin 2); *Opa1* (optic trophy 1, mitochondrial); *Ppargec1a* (peroxisome proliferator-activated receptor gamma coactivator 1); *P53* (tumor protein 53); *Slc25* (solute carrier family 25); *Sh3glb1* (SH3-domain GRB2-like endophilin B1); *Sod* (superoxide dismutase); *Timm* (translocase of inner mitochondrial membrane); *Tomm* (translocase of outer mitochondrial membrane); *Ucp* (uncoupling protein).

## Data Availability

All data generated or analyzed during this study are included in this article and its supplementary material files. Further inquiries can be directed to the corresponding author.

## Supplementary Materials

The following supporting information can be downloaded at: https://www.mdpi.com/article/10.3390/vision7010020/s1, Figure S1: Representative H&E-stained images of retinas from 21-day old rats exposed to 50/12% O_2_ intermittent hypoxia (IH) (panels A-C) and 21/12% O_2_ IH (panels D-E); Figure S2: Representative H&E-stained images of retinas from untreated 21-day old rats exposed to 50/12% O_2_ intermittent hypoxia (IH) at *P*14 (panel A), 21/12% O_2_ at *P*14 (panel B), 50/12% O_2_ at *P*21 (panel C), and 21/12% O_2_ at *P*21 (panel D); Figure S3: Representative image showing immunoreactivity of hypoxia inducible factor (HIF)_1α_ (panels A, B, E, F, I, J) and vascular endothelial growth factor (VEGF, panels C, D, G, H, K, L) in the retinal sections from groups supplemented with CoQ10 in RA (panels A and C, respectively), 50/12% O_2_ IH (panels E and G, respectively), and 21/12% O_2_ IH (panels I and K, respectively), or those supplemented with fish oil in RA (panels B and D, respectively), 50/12% O_2_ IH (panels F and H, respectively), and 21/12% O_2_ IH (panels I and K, respectively); Figure S4: Effects of neonatal IH on retinal (panels A and C) and choroid-RPE (panels B and D) hydrogen peroxide (H_2_O_2_) and superoxide dismutase (SOD) levels in untreated neonatal rats on postnatal day 14 (*P*14) and *P*21; Figure S5: Effects of neonatal IH on retinal (panels A and C) and choroid-RPE (panels B and D) catalase and glutathione levels in untreated neonatal rats on postnatal day 14 (*P*14) and *P*21.
