# Supplementary material for: Coenzyme Q10 and Fish Oil Supplementation for Reducing Retinal Oxidative Stress in a Rat Model
Source: Vision (Basel). 2023 Mar 11;7(1):20. doi: 10.3390/vision7010020 (PMC10052001; doi:10.3390/vision7010020)
Supplement: Supplementary file 1 [file vision-07-00020-s001.zip › vision-1960400-supplementary.pdf]

Siddiqui F et al.

Supplemental Figures

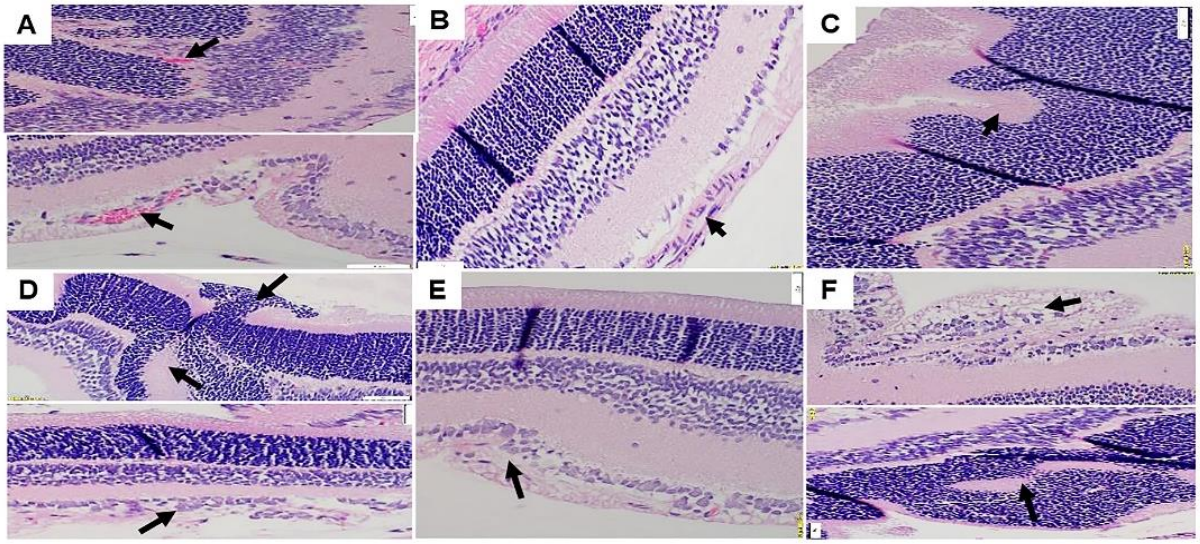

Figure S1. Representative H&E stained images of retinas from 21-day old rat exposed to 50/12% O_2 intermittent hypoxia (IH, panels A-C), and 21/12% O_2 IH (panels D-E). Animals were supplemented with olive oil (OO, panels A and D), coenzyme Q10 (CoQ10, panels B and E), or fish oil (panels C and F). Images are 40X magnification and the scale bar is 20 μM. Arrows show hemorrhage, and major abnormalities in the retinal layers.

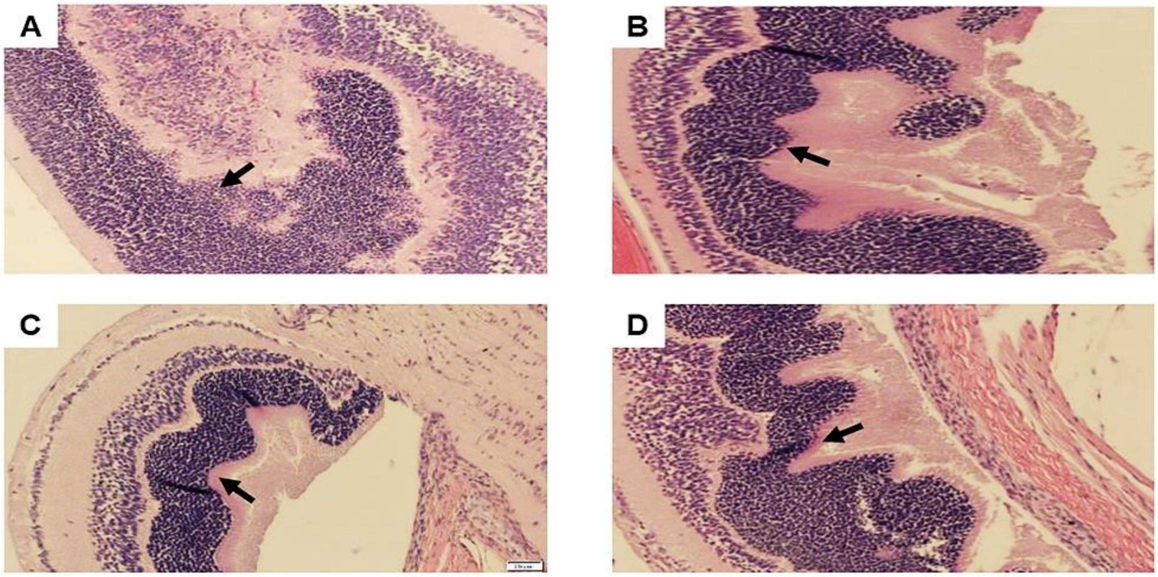

Figure S2. Representative H&E stained images of retinas from untreated 21-day old rat exposed to 50/12% O_2 intermittent hypoxia (IH) at P14 (panel A) 21/12% O_2 at P14 (panel B), 50/12% O_2 at P21 (panel C), and 21/12% O_2 at P21 (panel D). Images are 40X magnification and the scale bar is 20 μM. Arrows show major abnormalities in the retinal layers in both IH groups.

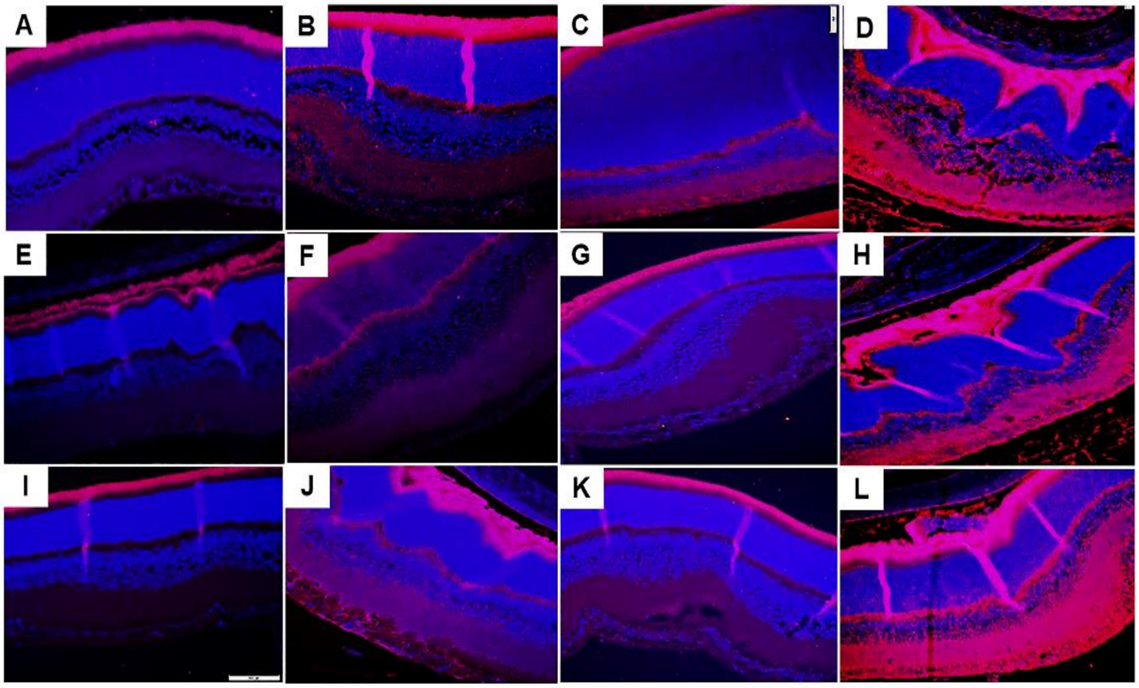

Figure S3. Representative image showing immunoreactivity of hypoxia inducible factor $(HIF)_{1\alpha}$ (panels A, B, E, F, I, J) and vascular endothelial growth factor (VEGF, panels C, D, G, H, K, L) in the retinal sections from groups supplemented with CoQ10 in RA (panels A and C, respectively), 50/12% O_2 IH (panels E and G, respectively), and 21/12% O_2 IH (panels I and K, respectively); or fish oil in RA (panels B and D, respectively), 50/12% O_2 IH (panels F and H, respectively), and 21/12% O_2 IH (panels I and K, respectively). CoQ10 decreased $HIF_{1\alpha}$ in RA (panel A), 50/12% O_2 IH (panel E), and 21/12% O_2 IH (panel I), and VEGF in RA (panel C), 50/12% O_2 IH (panel G), and 21/12% O_2 IH (panel K) compared to fish oil. Corresponding quantitaive analysis is presented in Table 1. Images are 40X magnification (scale=20 μM).

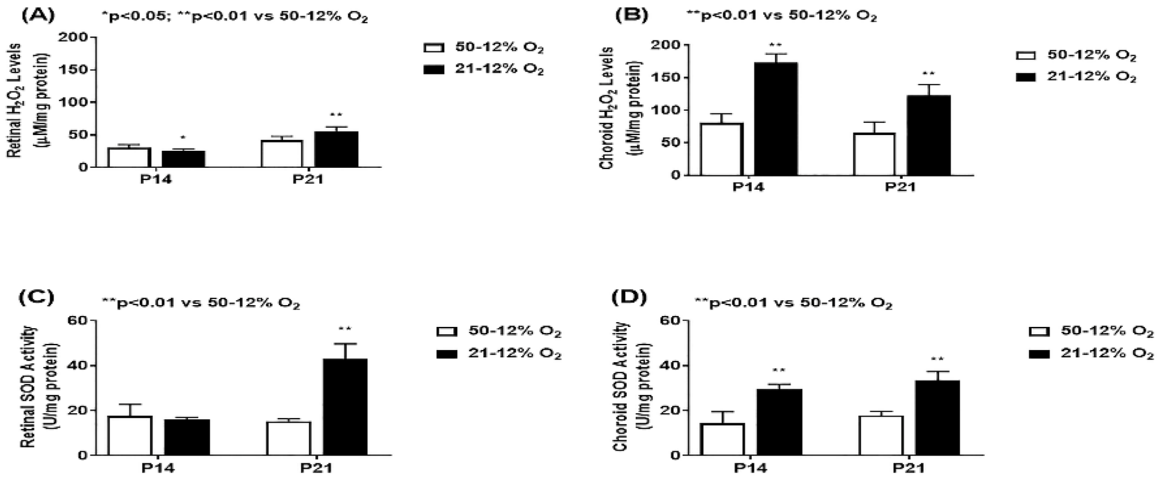

Figure S4. Effects of neonatal IH on retinal (panels A and C) and choroid-RPE (panels B and D) hydrogen peroxide (H_2O_2) and superoxide dismutase (SOD) levels in untreated neonatal rats on postnatal day 14 (P14) and P21. The white panel represents the animals exposed to 50/12% O_2 IH and the black panel represents the animals exposed to 21/12% O_2 IH. In the retina, H_2O_2 (panel A)

and SOD (panel C) levels were higher with 21/12% O_2 IH at P21. In the choroid-RPE, H_2O_2 (panel B) and SOD (panel D) were higher at P14 and P21. Data are expressed as mean ± SEM. *n* = 6 samples/group.

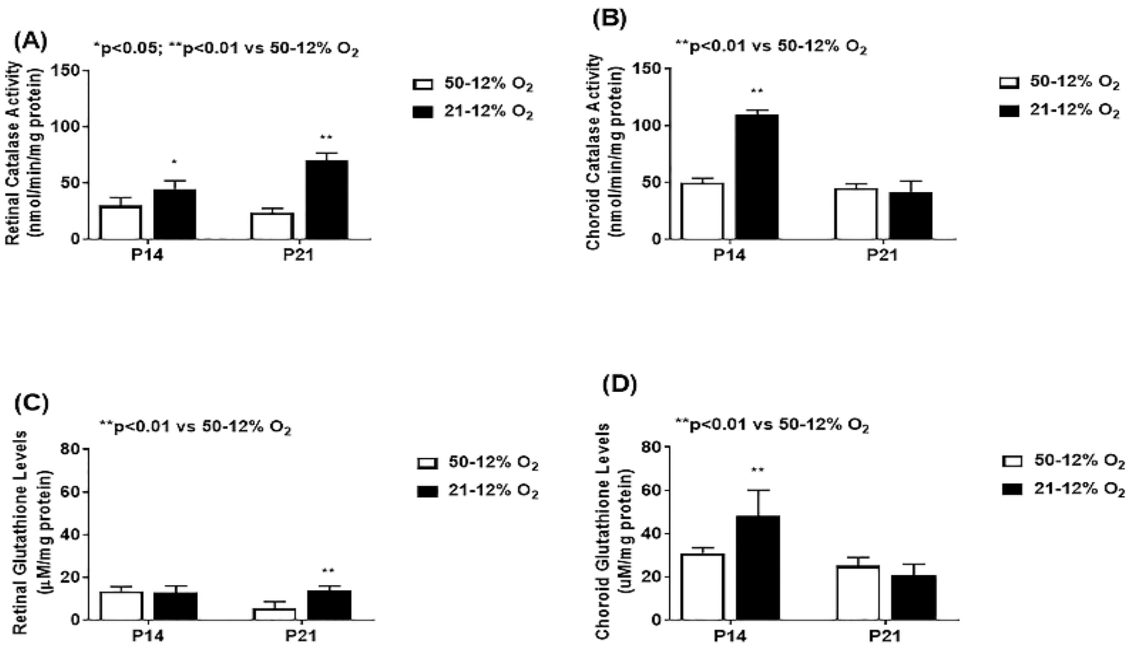

Figure S5. Effects of neonatal IH on retinal (panels A and C) and choroid-RPE (panels B and D) catalase and glutathione levels in untreated neonatal rats on postnatal day 14 (P14) and P21. The white panel represents the animals exposed to 50/12% O_2 IH and the black panel represents the animals exposed to 21/12% O_2 IH. In the retina, catalase levels (panel A) were higher with 21/12% O_2 IH at P14 and P21, while glutathione was increased at P21 (panel C). In the choroid-RPE, catalase (panel B) and glutathione (panel D) levels were higher at P14 only. Data are expressed as mean ± SEM. *n* = 6 samples/group.
